# Atherosclerotic Cardiovascular Disease: Risk Assessment, Prevention and Treatment Strategies

**DOI:** 10.3390/jcdd9120460

**Published:** 2022-12-14

**Authors:** Jernej Jeras, Sabina Ugovšek, Andreja Rehberger Likozar, Miran Šebeštjen

**Affiliations:** 1 Community Health Centre Ivančna Gorica, 1295 Ivančna Gorica, Slovenia; 2 Faculty of Medicine, University of Ljubljana, 1000 Ljubljana, Slovenia; 3 Department of Cardiology, University Medical Centre Ljubljana, 1000 Ljubljana, Slovenia; 4 Department of Vascular Diseases, University Medical Centre Ljubljana, 1000 Ljubljana, Slovenia

Despite enormous advances in both surgical and pharmacological treatment, cardiovascular diseases are still the most common cause of morbidity and disability in the western world. In this year’s Special Issue “Atherosclerotic Cardiovascular Disease: Risk Assessment, Prevention and Treatment Strategies” of *JCDD*, we aimed to include topics that cover early detection of more at-risk patients, the search for markers and factors that predict the outcome of the disease as well as surgical treatment and pharmacological treatment of atherosclerotic cardiovascular diseases and their consequences.

In their research on apparently healthy women, Mazgelyte and colleagues [1] showed that the increased concentration of stress-related steroid hormones in the hair is associated not only with post-metabolic risk factors, but also with the overall risk of future cardiovascular events, as calculated by the SCORE2 risk prediction algorithm. In patients with ischemic heart disease, a study by Birkl and colleagues [2] showed that coronary computed tomography angiography is an excellent gatekeeper in patients with suspected obstructive coronary artery disease before more invasive coronary angiography. Our first research study investigated the impact of proprotein convertase subtilisin/kexin type 9 (PCSK9) inhibitors in patients after myocardial infarction with higher-than-recommended LDL cholesterol levels and highly increased lipoprotein(a) values on the gene expression of markers of coagulation, fibrinolysis, inflammation, and angiogenesis, which all play a very important role in the atherosclerotic process. Our results underpin the previous knowledge by demonstrating the importance of these markers, in particular interleukin-1β, vascular endothelial growth factor-A and tissue factor (TF) [3]. Our second study investigated three polymorphisms, namely rs1800947 in C-reactive protein, rs1800629 in tumor necrosis factor-α (TNF-α), and rs1800795 in interleukin 6 and their association with their corresponding plasma levels after treatment with PCSK9 inhibitors in the same cohort of patients. We showed that levels of these inflammatory markers did not change after treatment with PCSK9 inhibitors. However, after 6 months of treatment with PCSK9 inhibitors, the IL6-174CC genotype was associated with a significant difference in interleukin 6 levels, indicating the possible role of this genotype in the regulation of inflammation [4]. In addition to drug therapy, regular physical activity is very important for patients with ischemic heart disease. In their research, Jug and colleagues [5] determined whether aquatic exercise improves heart rate variability (HRV) parameters compared with land-based training. Aquatic exercise training was associated with a significant change in selected HRV parameters. Their results provide evidence on the distinct impact of aquatic exercise training on cardiac autonomic modulation; on the other hand, the specific pattern of HRV changes may suggest distinct cardiovascular benefits of water-based exercise modalities in patients with coronary artery disease.

In patients with symptoms of peripheral vascular disease, the ankle–brachial index, even when in the range of normal values, has been shown to be a good predictor of future cardiovascular events. Peltonen and his colleagues [6] discovered that the lower ankle-brachial index, the greater the cardiovascular risk. In their research, Pelicon and colleagues [7] compared the group of patients who underwent endovascular femoropopliteal revascularization and received antiaggregating therapy only, and those that, due to other comorbidities, also received anticoagulant therapy in a therapeutic dose. They hypothesized the difference in overall mortality, worsening of peripheral vascular disease or frequency of amputations due to vascular causes. However, they found no differences in the above-mentioned end points, nor in the risk of bleeding. Bleeding, although less intensive compared with treatment with vitamin K antagonists, is also one of the major side effects of the new anticoagulants. This is of particular importance in emergency situations in bleeding patients in whom transfusion or the use of a neutralizing agent may be considered. Thus, the aim of Božič et al.’s [8] research was to evaluate the biological variation in rotational thromboelastometry (ROTEM), where clotting can be induced by TF in high (Extem) or low concentrations (LowTF), as well as prothrombin time, activated partial thromboplastin time, and anti-Xa in patients receiving rivaroxaban. Their knowledge of these biological variation components will help to establish the appropriate objective analytical performance specifications and calculate the reference change value to decide whether there is a significant difference between the two test results from the same individual.

Heart failure (HF) is a disorder in which the heart is unable to pump blood to the body at a rate commensurate with its needs, or can do so only at the cost of high filling pressures [9]. Despite such a universal definition, there are many causes and forms of HF. Endothelial dysfunction is common to both ischemic and non-ischemic HF. Both impaired endothelial function and reduced CD34+ cell concentrations, are hallmarks of both, ischemic heart disease and heart failure [10,11]. The study of Ugovšek et al. [12] showed that increased levels of TNF-α were associated with impaired endothelial function as well as with a lower number of CD34+ cells after stimulation with granulocyte colony stimulating factor. Hence, we suggest that high TNF-α levels are one of the main factors affecting both the endothelium and the bone marrow function in patients with HF, regardless of the cause of HF. Conduction disorders, the most common of which is atrial fibrillation (AF), are very often present in patients with HF and affect both quality of life and mortality. Atrio-ventricular node ablation (AVNA) with subsequent permanent pacemaker implantation provides a definite rate control and represents an alternative therapeutic approach in patients with symptomatic AF and rapid ventricular rate, refractory to optimal medical treatment, or catheter ablation [13]. Ivanovski et al. [14] aimed to compare the clinical outcomes of biventricular (BiV) pacing, His bundle Pacing (HBP) and left bundle branch pacing (LBBP) in HF patients with symptomatic AF and narrow QRS who underwent AVNA. Their results showed first, that AVNA combined with conduction system pacing (HBP and LBBP) was feasible and safe in high percentage of patients with rate refractory AF and HF. Second, they showed that the conduction system pacing was associated with superior clinical and echocardiographic improvement compared with BiV pacing. Heart failure with preserved ejection fraction (HFpEF) is very common; however, unlike HF with reduced ejection fraction (HFrEF), there is no evidence-based effective treatment. Therefore, among patients with HFpEF, it is more important to identify those at high risk and to treat them more aggressively. According to the findings of Ksela et al. [15], heart rate turbulence (HRT) and variability (HRV) have the ability to differentiate individuals with HFpEF who are at the greatest risk of unfavorable outcomes.

Acute kidney injury is one of the most common complications after coronary artery bypass grafting. Minor subclinical creatinine changes have been shown to be prognostic for predicting acute kidney injury in the period of 6 to 12 h after cardiac surgery [16]. Kalisnik et al. [17] found that the addition of neutrophil-gelatinase-associated lipocalin and cystatin C to the model in addition to creatinine, provides a better prediction of cardiac surgery-associated acute kidney injury compared with any biomarker alone as early as two hours after the termination of the cardiopulmonary bypass.

In addition to research articles, this issue of *JCDD* also includes two review articles covering two very common pathologies in the modern cardiovascular medicine. The stenosis of the aortic valve is a frequent, most commonly degenerative process that is becoming increasingly prevalent with the ageing population [18]. Steblovnik et al. [19] provided an overview of the technical aspects of transcatheter implantation of the aortic valve, which is indicated in patients with high risk for a classic operative procedure. Despite the enormous progress in the treatment of HF in recent years, the number of these patients is increasing rapidly, and new treatment options are being sought. Knowledge about stem cell treatment options has advanced greatly in the recent years. Banovic et al. [20] have accurately revised the current knowledge of this field and highlighted contemporary challenges and dilemmas in clinical aspects of stem cell and regenerative therapy in patients with chronic ischemic and non-ischemic HF.

In this Special Issue of *JCDD*, “Atherosclerotic Cardiovascular Disease: Risk Assessment, Prevention and Treatment Strategies”, we have presented several new findings in the field of risk assessment, diagnosis, and treatment of various cardiovascular diseases. As always, this has opened a lot of questions, which we will be able to answer with the results of new research.
